# BIM-based digital platform and risk management system for mountain tunnel construction

**DOI:** 10.1038/s41598-023-34525-w

**Published:** 2023-05-10

**Authors:** Naifei Liu, Desai Guo, Zhanping Song, Shiming Zhong, Ruoqi Hu

**Affiliations:** 1 School of Civil Engineering, Xi’an University of Architecture and Technology, Xi’an, 710055 People’s Republic of China; 2 Key Laboratory of Geotechnical and Underground Space Engineering, Xi’an University of Architecture and Technology, Xi’an, 710055 Shaanxi Province People’s Republic of China; 3 College of Civil Engineering, Hunan University, Changsha, 410082 People’s Republic of China

**Keywords:** Civil engineering, Information technology

## Abstract

During the construction of mountain tunnels, there are often various intricate and mutable potential hazards, the management and control of which are crucial to ensuring the safety of such construction. With the rapid advancement of engineering information technologies, including Building Information Model (BIM), the internet, big data, and cloud computing, dynamic management of mountain tunnel construction will inevitably become a prevailing trend. This paper proposes a new digital approach to realize the informatization and visualization of risk management in mountain tunnel construction, by combining monitoring measurement with advanced geological prediction based on BIM technology. The proposed approach suggests a BIM-based digital platform architecture for mountain tunnel construction, which is comprised of five layers—basic, model, data, application, and user. The integration of these five layers can realize risk management information during the construction of mountain tunnels. In addition, a set of dynamic risk management systems, including risk monitoring, identification, and assessment, can be established based on the digital platform. The digital platform and dynamic risk management system proposed in this paper have certain advantages in the construction of mountain tunnels, providing a new and significant way for the management of safety risks in such construction projects.

## Introduction

Currently, China holds the title of having the largest scale, number, and most complex geological conditions and structural forms in the tunnel and underground engineering, with the fastest development of construction technology worldwide^1^. As of the end of 2020, China's railway mileage had reached 145,000 km, with 16,798 railway tunnels in operation, spanning approximately 19,630 km. In addition, there are 21,316 operational tunnels on highways above grade, with a total length of about 21,999 km^2^. However, during the construction process of mountain tunnels, complex geological conditions are inevitably encountered, and various serious accidents often occur, leading to significant social impacts and immense economic losses^3^. Therefore, ensuring the safety and efficiency of mountain tunnel construction has become a vital goal in modern tunnel construction.

Mountain tunnel construction is a highly complex and potentially risky project. With the excavation of the tunnel, the geological conditions will be unstable and do not match the survey and design data, may cross the fault, fold layer, weak fracture zone, and may also encounter high ground stress soft rock, gas, karst and other adverse geology^4–6^. The design of the mountain tunnel is mainly based on the theory of the New Austrian Tunnelling Method (NATM). Different mountain tunnels surrounding rock stability, grade change degree, excavation method is different, and construction method is not fixed. The construction management mode of tunnel construction projects mostly adopts the Design–Bid–Building (DBB) mode, which limits the communication between different units and hinders the information flow^7^. If there is no complete risk control system in tunnel construction, it will bring great harm to the whole construction process. The existing methods are too dependent on experts in this field. Due to time constraints, experts cannot provide real-time guidance. If a collaborative risk management model is established, it will provide extremely effective help to solve the above problems, and the emergence of BIM technology provides strong technical support for the realization of this management model.

BIM technology has been widely used in many fields of civil engineering. According to the definition of BIM by the National Building Information Model Standards Project Committee, BIM refers to the digital representation of physical and functional characteristics of facilities^8^. In essence, BIM is a life-cycle shared database that includes the stage of project design, construction, operation management, and maintenance. It can effectively improve project progress and efficiency, control project cost, and reduce project risk^9^. However, due to the zonal distribution of tunnel engineering and the inseparable relationship between tunnel structure and geological body, it has a strong geological correlation^10^. Therefore, compared with construction engineering, the application of BIM in tunnel engineering has certain particularities. Complex geological conditions and project management mode may become the biggest bottleneck of BIM application in mountain tunnels.

Smooth blasting, bolting, and shotcrete support and monitoring measurement are the three core contents of the new Austrian tunneling method (NATM)^11^. The monitoring measurement data reflect the rock properties and state of the surrounding rock near the working face, and then the managers evaluate the stability and safety of the surrounding rock through these data to guide the field construction^12^. However, the data obtained by monitoring measurement is still unable to achieve visual overall collaborative management. Therefore, this paper also takes this as the starting point. On the one hand, this paper combines BIM technology with monitoring measurement and advanced geological prediction, two key parts of mountain tunnel construction, for the first time, and preliminarily proposes the digital platform architecture of mountain tunnels based on BIM technology. On the other hand, this paper introduces BIM technology and risk management into the field of mountain tunnel engineering for the first time and puts forward a whole set of dynamic risk management systems based on the digital platform. These are significant innovations in this paper. This paper provides a new technical approach for mountain tunnel construction and risk management, which is of great significance for safety and risk control during mountain tunnel construction.

The remaining parts of this paper are organized as follows: "Literature review" section gives literature reviews of the development of BIM technology and its application in tunnel engineering management and tunnel risk analysis. "Method" section introduces monitoring measurement, advanced geological forecast, and BIM technology. "The digital management platform" section introduces the framework of the digital management platform. "Risk management of mountain tunnel" section introduces the mountain tunnel dynamic risk management system. In "Discussion" section, we discuss the advantages, disadvantages, and prospects of digital platforms and dynamic risk management systems. In "Conclusions" section, conclusions are drawn.

## Literature review

### Development status of BIM technology BIM

In 1975, Dr. Chuck Eastman first proposed Building information modeling (BIM)^13^. BIM is to collect, analyze and apply all the data generated in the whole life cycle of the project. It has five characteristics: visualization, coordination, simulation, optimization, and drawing^14,15^. It is widely promoted and used because it meets the needs of social development. Driven by national policies, BIM ushers in a stage of rapid development. BIM has the following major development trends. First of all, the application field of BIM technology is expanding. At first, BIM technology is mainly used in the field of housing construction^16^, and then it is expanded to the fields of tunnel^17^, bridge^18^, transportation infrastructure^19^, underground utility tunnels^20^, hydropower engineering^21^, and so on. At present, researchers have applied BIM technology to teaching^22^, green buildings^23–25^, and road asset management^26^. Secondly, the integration of the project life cycle based on BIM. With the gradual improvement of the BIM standard system, BIM technology will play a role in the whole life cycle of the project^27^. Finally, the integration of BIM and other technologies. Researchers are constantly exploring possibilities for integrating more technologies with BIM, such as BIM + FE (Finite Element)^28,29^, BIM + AR (Augmented Reality)^30^, BIM + VR (Virtual Reality)^31^, BIM + GIS (Geographic Information System)^32^, BIM + 3D laser scanning technology^33,34^ and BIM + CV (Computer Vision)^35^.

### Application of BIM in tunnel engineering management BIM

Given the advantages of BIM technology, some scholars try to apply BIM technology to tunnel engineering management. Song et al. proposed a preliminary construction scheme for a tunnel engineering collaborative management platform based on BIM technology and analyzed the feasibility of platform development in depth^36^. Hegemann et al. constructed the position and direction completion documents of the tunnel TBM construction segment lining ring based on BIM and realized the connection between BIM and TBM data acquisition, the determination of geometric transformation, and the connection of semantic information^37^. Borrmann et al. combined BIM technology with GIS system to realize the visualization of the tunnel construction process^38^. Lee et al. combined BIM technology with GIS system to develop a tunnel maintenance management system and applied it to tunnel construction management, which proved the practicability and applicability of the system and had good prospects^39^. Ninić et al. combined numerical simulation with BIM technology proposed a new concept of numerical simulation based on BIM and developed a computational simulation system for urban mechanized tunnels based on a multi-level BIM model^40^. Yu et al. summarized the limitations of the traditional tunnel emergency management model-geometric network model, proposed a multi-purpose tunnel data model, and obtained the corresponding emergency response algorithm^41^.

### Application of BIM in tunnel construction risk

Some scholars have also done some research on BIM in tunnel construction risk management. Li et al. proposed an automatic safety risk identification process based on BIM and developed an automatic safety risk identification system^42^. The system is applied to a subway construction to verify the feasibility and effectiveness system. Zhang et al. proposed an innovative method combining BIM with an expert system to establish an integrated knowledge base composed of a fact base, rule base, and case base to solve the shortcomings of traditional tunnel construction safety risk identification process^43^. Du et al. found that BIM technology has poor information interoperability, and proposed an ontology-based information integration framework^44^. At the same time, she used the hierarchical clustering method to analyze the risk factors of shield tunnel surface subsidence and predicted potential construction damage and surface subsidence. Li et al. proposed the Safety risk identification system (SRIS) and early warning system (SREWS) for China’s metro construction based on the BIM platform and discussed the modeling process, methods, and key technologies of SRIS and SREWS in detail^45^. Liang and Liu used BIM technology and Internet of Things technology to analyze the construction of underground engineering safety risk early warning systems and realized real-time dynamic monitoring of underground construction safety risk early warning systems^46^. Based on the 3D-BIM model, Providakis et al. used the IFC standard as the bridge between BIM data and MATLAB grid division analysis tool to evaluate the risk of tunnel safety construction^47^.

### Research summary

At present, the application of BIM technology in engineering construction has developed rapidly, and some achievements have been made in tunnel construction. Meanwhile, the literature also fully demonstrates the use of BIM technology to assist mountain tunnel construction, which becomes an inevitable trend in the future. However, there are some shortcomings in the application of BIM in tunnel engineering. On the one hand, monitoring measurement and advanced geological prediction are always two key links in the process of mountain tunnel construction and risk management. At present, no one has combined BIM technology with monitoring measurement and advanced geological prediction. On the other hand, Most of the research on BIM technology combined with risk management belongs to the field of subway engineering, while the research in the field of mountain tunnel engineering is relatively few. In addition, BIM technology is the inevitable choice of the future construction industry, and tunnel construction cases involving BIM technology are increasing. By using the advantages of BIM technology, it is feasible to build a digital platform for mountain tunnels by combining BIM technology with monitoring measurement and advanced geological prediction and to introduce BIM technology and risk management into the field of mountain tunnel engineering.

## Method

In the construction of mountain tunnels, monitoring measurement and advanced geological prediction are two more important parts. From the beginning to the end of the project, a large amount of monitoring measurement and advanced geological prediction data information will be generated^48^. The traditional data and information management modes are all independent management modes. The proposal of a digital management platform means the birth of a new management mode, which makes it possible to comprehensively manage mountain tunnel engineering monitoring and measurement and advanced forecast information^49^. The engineering information reflected by the BIM model is the most objective basis for judging the safety risk of underground engineering. Specifically, the construction safety risk of underground engineering is directly related to the construction technology, construction method, and construction organization of underground engineering. Based on this advantage, this article combines BIM technology to build a digital management platform for mountain tunnel construction and proposes a set of risk management systems. Based on the digital management platform, the mountain tunnel construction data information can be managed, and a large amount of engineering data information can be extracted and analyzed, which has extremely important significance in information integration and information sharing.

**Figure 1:**
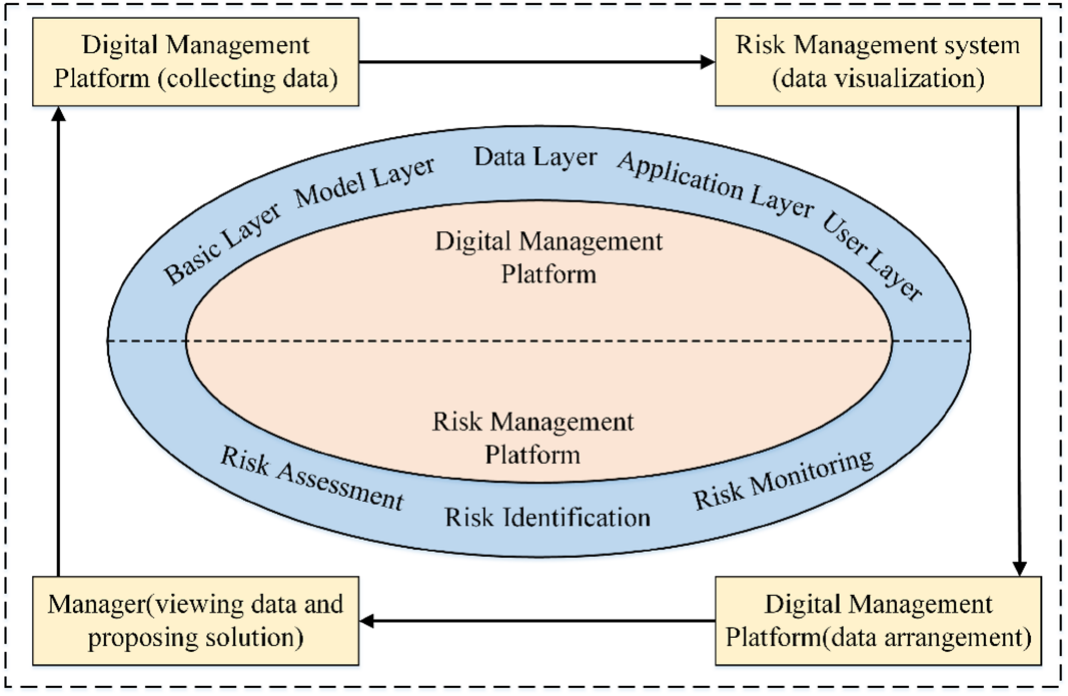
Technology roadmap about risk management platform and risk management system.

The risk management system can solve communication problems between managers. This system uses the BIM model to access different levels of management work according to different user roles. The system is an internet platform that utilizes BIM model illustration with different levels of access to work that depends on user roles. When the information is updated in the system, the server automatically sends the information to the relevant managers. Figure 1 is the risk management platform and risk management technology roadmap.

### Monitoring measurement

Monitoring measurement is a powerful tool to ensure the safety of tunnel construction. It involves observing and recording the surface condition of the shotcrete layer after the primary lining of a tunnel is completed, and describing the deformation of the surrounding rock. The purpose of monitoring measurement is to properly classify the settlement rate of surrounding rock and evaluate its stability through the description of the geological and lining conditions of the surrounding rock. In addition, monitoring measurement is performed to monitor the tunnel vault settlement and surrounding convergence displacement to confirm whether the surrounding rock is stable and whether the lining is effective. This helps guide the construction, prevent collapse, and ensure construction safety. In monitoring measurement, surrounding rock pressure, vault displacement, peripheral convergence, and surface settlement must be measured. A large amount of monitoring data is generated from the beginning to the completion of a tunnel project, so it is very important to manage and analyze monitoring data scientifically and effectively. The BIM model can be used as the carrier of tunnel monitoring information storage and display due to its integration, information completeness, and other characteristics.

The in-depth integration of BIM technology and monitoring measurement technology is studied. The monitoring measurement points are created with BIM modeling software and given corresponding attributes such as coding positions. The monitoring data and early warning information of each measurement point is linked to the corresponding measurement point model through the Internet of Things technology. At the same time, the key information extracted from the photo plane graphic elements were mapped to the 3D tunnel spatial structure through the graphics processing technology and the tunnel disease elements were mapped to the spatial model of the tunnel structure to realize the 3D visual representation of the tunnel disease. Data Mining is used to analyze and interpret all the data obtained from the monitoring measurement. When the monitoring data exceeds the preset threshold, it will automatically alarm, and the dangerous area will be marked with different colors in the BIM model, to realize the tunnel vault settlement. The visual monitoring and warning effect of peripheral convergence and headroom change can finally achieve the dynamic visual display of tunnel monitoring and measurement data according to the actual excavation progress of the tunnel, to guide the tunnel site construction more accurately and ensure the safety of the tunnel construction.

#### Arrangement of monitoring points and considerations

The arrangement of monitoring points varies according to the site conditions of the project. In general, however, the spacing of monitoring points should follow the following principles: at most 5 m for Class V surrounding rock, at most 30 m for Class IV surrounding rock, 30–50 m for Class III surrounding rock, and 50–100 m for Class II surrounding rock. The measuring points at different sections should be placed at the same position of a tunnel and checked by monitoring personnel regularly. For monitoring, the monitoring points should be, if possible, kept away from material transportation and stacking sites and intensive operation areas to reduce the errors caused by blocking the points in field observations and also to avoid disturbance of the monitoring points. Surface monitoring points should be set out in a hidden manner to prevent them from being damaged by human or animal activities.

#### Data processing process

After site monitoring points are arranged, field monitoring is carried out, and the monitoring data is processed and reported to the project participants. The monitoring data collected at each monitoring point is used to develop a curve showing the variation of surrounding rock shape with time, and regression analysis is performed. The curve provides an insight into surrounding rock deformation at each measuring point. At the points where serious deformation is observed, it is necessary to carry out measurements several times and especially mark the points. Commonly used regression functions include logarithmic functions, exponential functions, and hyperbolic functions, see Table 1. These regression functions can be used to describe the pattern where the surrounding rock shape varies with time and to predict maximum deformation. The monitoring data is input into the tunnel models, and each party in a tunnel project can check the deformation of the surrounding rock of the tunnel at any time. The deformation pattern at each monitoring point facilitates guidance on construction by predicting the stability time at the current point.

**Table 1:** Comparison and evaluation of prediction accuracy of four kinds of geophysical prediction methods.

Function	Introduce variable	Parameters
*u* = *a* lg(1 + *t*)	*x* = *a*lg(1 + *t*)	*a*
*u* = *a e*-*b*/*t*	*x* = 1/*t*, y = ln*u*	*a*, *b*
*u* = *t*/(*a* + *b t*)	*y* = 1/*u*, *x* = 1/*t*	*a*, *b*

### Advanced geological prediction

Although the design unit has surveyed the geological condition of the tunnel before construction, it is impossible to know the situation about the surrounding rocks before tunnel excavation, and in addition, with the deepening of excavation, the surrounding rocks of the tunnel may change, such as weathering and water seepage. Thus, the construction plan can be developed better only in the event of the advanced geological prediction of the surrounding rocks’ geological conditions and the real-time mastery of the geological prediction information.

The geological conditions that can be detected by tunnel geological prediction include karsts, voids, fault structures and fault fracture zones, sizes and fillings of joint fractures, groundwater storage states, locations of water gush, water volumes, etc. Table 2 lists the commonly used advanced geological prediction methods, and their advantages and disadvantages^50^. To improve the accuracy of geological prediction, it is necessary to analyze it in an all-around way by combining the measurement results of various advanced geological prediction methods.

**Table 2:** Comparison and evaluation of prediction accuracy of four kinds of geophysical prediction methods.

Geophysical method	Accuracy appraisement				
	Fault	Fractured rock mass	Water situation	Karst caves (dry)	Weak rock
TSP	A	B	C	C	B
Geological radar	A	A	C	B	C
Transient electromagnetic method	D	D	A	D	D
Advanced horizontal drill	A	A	C	A	B

A, B, C, and D represent a geological situation respectively. Compared with other methods, the method is high, relatively high, fairly low, and low in terms of accuracy.

The BIM technology is used to deeply integrate the TTP advanced drilling information and other advanced geological prediction information with the BIM model of tunnel geology, and the color transparency of fault water-rich legend warning label and other technical means are used to realize various geological conditions in front of the tunnel excavation face The 3D visualization display and reminder of the hydrology and surrounding rock condition can provide auxiliary decision-making and information traces for the grade change of surrounding rock, and provide effective evidence for the actual change of the later engineering At the same time, combined with the BIM model of tunnel geology, the risk level and risk degree of the tunnel are automatically identified according to the geological conditions such as the grade of tunnel surrounding rock, so as to realize the visualization of safety risk assessment, guide the operation of tunnel excavation ahead, and ensure the safety of tunnel excavation.

### Advantages and objectives of BIM technology

#### BIM benefits

**Figure 2:**
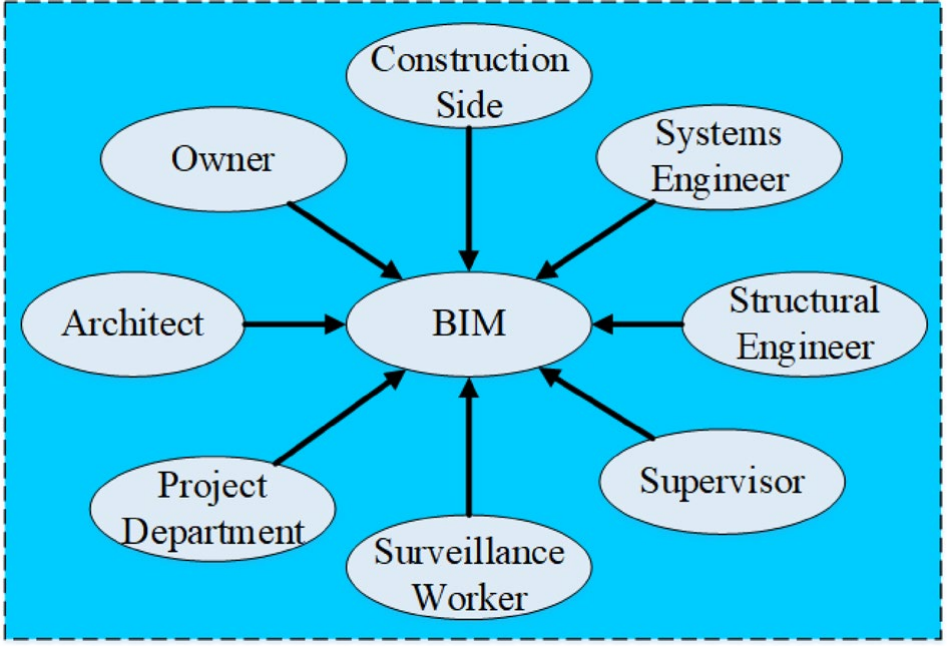
BIM technology characteristics.

In the past, construction works heavily relied on drawings. This reduces their efficiency and increases their workload as staff from all sectors could communicate with each other only through drawings. With the development of BIM technology, new BIM-based integrated management methods for design, construction, operation, and maintenance have greatly improved work efficiency. BIM technology connects project owners, design units, construction units, supervision units, and other project participants to provide an effective management tool for information-based engineering design and scientific construction^51^. Figure 2 is a schematic diagram of the characteristics of BIM technology.

Given the advantages of BIM technology, some scholars try to apply BIM technology to the risk management of mountain tunnel engineering. Advantages of the application of BIM technology in tunnel engineering include (1) Technical applicability. The BIM model of the mountain tunnel project contains all the information about the whole construction cycle, which can make the process of risk management more comprehensive and accurate. BIM will provide strong data support for project risk managers. (2) Industrial applicability. China's investment in the urban subway industry is on the rise every year. However, underlying the rapid development and huge investment, a large number of unnecessary economic losses are caused every year due to various mismanagement of subway projects. (3) Environmental applicability. Although BIM technology was introduced late in China with a low level of promotion and popularization relatively, the technology is gradually attracting the attention of the government and related departments. Whether from the perspectives of BIM technology promotion speed, or the approval of the technology by relevant departments, the environment for BIM technology promotion and its rapid development has been well developed. Consequently, it is feasible for this research to apply BIM technology to risk management of mountain tunnel construction.

#### Application objectives

The goal of BIM application in tunnel construction is to realize information-based and visual monitoring measurement and geological prediction for tunnel engineering. This consists of monitoring the conditions of the tunnel face of the excavation section and surrounding rocks, predicting the conditions of surrounding rocks in front of the tunnel face and abnormal tunnel conditions, and evaluating and analyzing possible risks to make tunnel construction efficient and safe.

## The digital management platform

**Figure 3:**
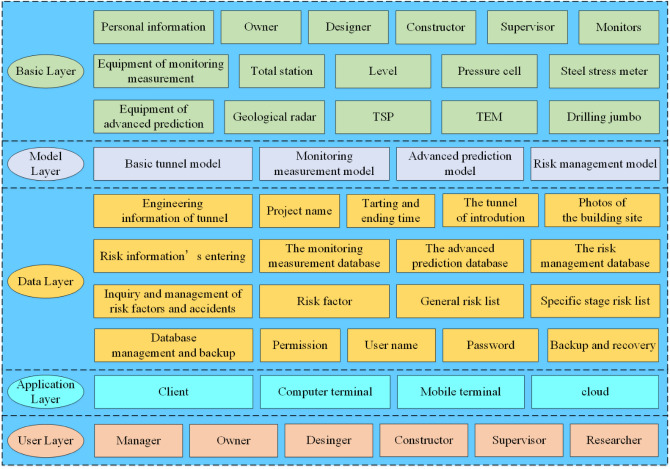
Schematic diagram of BIM management platform.

The digital management platform uses the typical five-layer architecture design, i.e., basic layer, data layer, model layer, application layer, and user layer. Figure 3 shows the architecture diagram of the digital management platform. The basic layer is mainly responsible for people's information and hardware equipment of the whole system. It includes some pieces of equipment for monitoring measurement and advanced geological prediction. The model layer is mainly responsible for the optical storage of the model, including the basic tunnel model, monitoring measurement model, advanced geological prediction model, risk management model, etc. The data layer is mainly responsible for the structured storage of data. The data should be stored separately to prevent confusion. The application layer is a user-oriented client program that provides fast access to users and systems. To meet the different needs of users, three different applications, including a computer terminal, mobile terminal, and cloud, should be established. Users can use these applications to achieve data query, visual browsing, data analysis, structural analysis, etc. The user layer is the group that uses the dynamic risk management system of the mountain tunnel. The groups include designers, researchers, supervising engineers and constructors, etc. The construction of the basic layer, model layer, and data layer is described in detail.

### Basic layer

To manage the problems of responsibility in management, we propose to build the basic resource information module. Basic resource information is the basis of tunnel management. It mainly includes people with information and hardware equipment. Through this basic information module, the manager can know the operation of equipment in the tunnel, and the manager classifies the equipment and manages the equipment. At the same time, the manager can also locate all the equipment by checking the account of the equipment. The manager can manage the basic information of the construction personnel through the basic resource information module. The manager can arrange the tunnel construction plan, and in case of an emergency, construction personnel can be contacted quickly. The probability of risk will be reduced and management efficiency will be improved. The closed information feedback control loop provides efficient means for managers to manage all types of equipment and lots of construction information.

### Model layer

**Figure 4:**
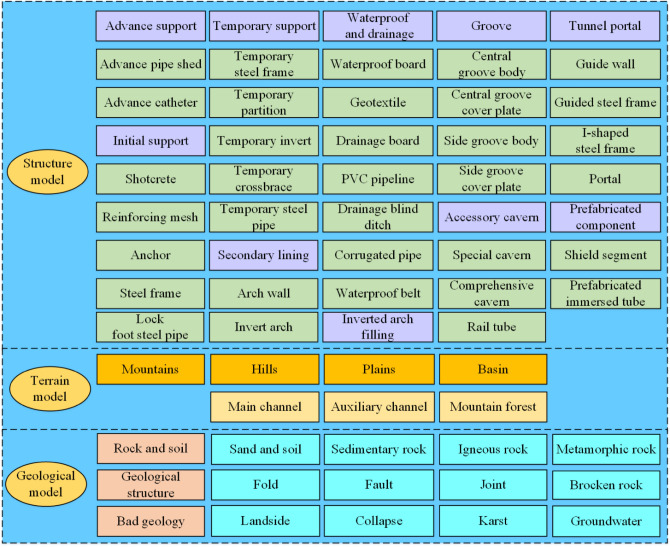
BIM model information.

The accuracy and effectiveness of the data layer are affected by the acquired security risks, so it is necessary to accurately model. According to the NATM, we break down the tunnel structure into basic components. When we model, we should input the basic information of components, and build a BIM model component library about mountain tunnels. Any disunity of the name may cause errors in the information transfer process, from the name of components to models even the name of project files. It is very serious when it has islands of information and the value of informatization is reduced. To make BIM technology exert the maximum effect in mountain tunnel construction, at the same time to carry out unified management of tunnel construction, a set of systemized naming criteria is required. The component of the BIM model can be divided into advance support, initial support, secondary lining, waterproofing and drainage and inverted arch filling, etc. The coding rules can be combined in alphabetical Figure.s. For example, the advanced small conduit’ coding is A-1-01-001. "A" is a first-level code, which represents the first-class classification of a small advanced conduit-component library. "1" is a second-level code, which represents the second-class classification of small advanced conduit—advance support. "01" is a third-level code, which represents the component of this small advanced conduit. "001" is a fourth-level code, which represents the location of this small advanced conduit, the manager can easily find the source of the danger. We use Revit to build the basic model of the mountain tunnel. It includes a 3D geological model, a 3D terrain model, and a main tunnel structure model, as shown in Fig. 4.

During the modeling process, we should sign the local monitoring point and import information about the component. So that managers can observe the monitoring site. During the modeling process, the information on mountain terrain, tunnel length, height clearance, buried depth of tunnel top, and the thickness of the secondary lining is generated and gradually gathered. The information lays a good foundation for calculating quantities and costs based on the model in a later stage. The safety risks of cracks or even collapse, pipeline leakage, or fracture in the tunnel, as well as the risks affecting the safety of existing structures, can all be shown in the BIM model.

### Data layer

The safety risk of mountain tunnels is related to many factors, such as project information, construction plan, tunnel location information, and surrounding environment information. The data and information involved in the tunnel construction are of various types and complex forms, which will be constantly updated with the progress of mountain tunnel construction^52^. Two types of people use the tunnel construction risk database. One is the managers. They record, and statistically analyze the mountain tunnel construction history accidents in the database, and they manage construction through these data. With these ways, managers can accurately obtain the basic information that they need in the future of the mountain tunnel construction, and they can conduct risk identification and risk assessment. The other is the students and technicians who use the system. They can know some construction methods of NATM, different risk factors in different construction phases, and historical data on this tunnel.

Based on it, there are four main functions of the mountain tunnel construction risk database. The first is the engineering information about the tunnel. It includes the project name, starting and ending time, the tunnel introduction and some pictures of the construction, etc. The second is risk information entering, Statistics, and analysis. According to the differences in data types and the division of functional modules in the application layer of the management platform, the data layer can be divided into three databases, namely, the monitoring measurement database, the advanced geological prediction database, and the risk management database. The tunnel model information database includes initial mixing, second lining, inverted arch, and monitoring point position in the tunnel. The monitoring measurement database includes several sub-databases in terms of surrounding rock pressure, vault settlement, surface settlement, convergent deformation, etc., and the advanced geological prediction database includes tunnel seismic prediction (TSP), geological radar, TEM (transient electromagnetic methods) and advanced horizontal drilling. The processed monitoring measurement and advanced geological prediction data are stored in the risk management database. The monitoring measurement and advanced geological prediction are introduced in "Method" section. The risk management process is described in detail in "Risk management of mountain tunnel" section. The third is the inquiry and management of risk factors and accidents. It includes risk factors and events of mountain tunnel construction. It gives a list of one stage of construction and specific risk. The fourth is some functions that can manage and back up data. The database system should give different permissions to different users. It can classify different managers, including user names, passwords, permission, etc.

### Application layer

The application layer is the client program oriented towards users, which provides a user-friendly interface for accessing the system. To meet the diverse needs of users, it is necessary to establish three different types of applications: computer-based, mobile-based, and cloud-based. Users can use these applications to achieve functions such as data query, visualization browsing, data analysis, and structural analysis of mountain tunnels.

### User layer

The user layer refers to the group of individuals who use the Mountain Tunnel Dynamic Risk Management System, including owners, designers, construction personnel, supervisors, and researchers. Administrators can set permissions for each group and set their account passwords. Each management group is classified and all data is categorized. The design data includes geological conditions, advanced forecasting, concrete strength, and mechanical facilities. Construction data includes BIM construction simulation, monitoring data, and mechanical equipment status. Research data includes rock pressure, pressure, water content, porosity, steel arch stress, and pressure. Supervisors mainly compare this data against design requirements based on their experience and standards. Owners manage all data and issue next-step commands based on construction progress.

## Risk management of mountain tunnel

**Figure 5:**
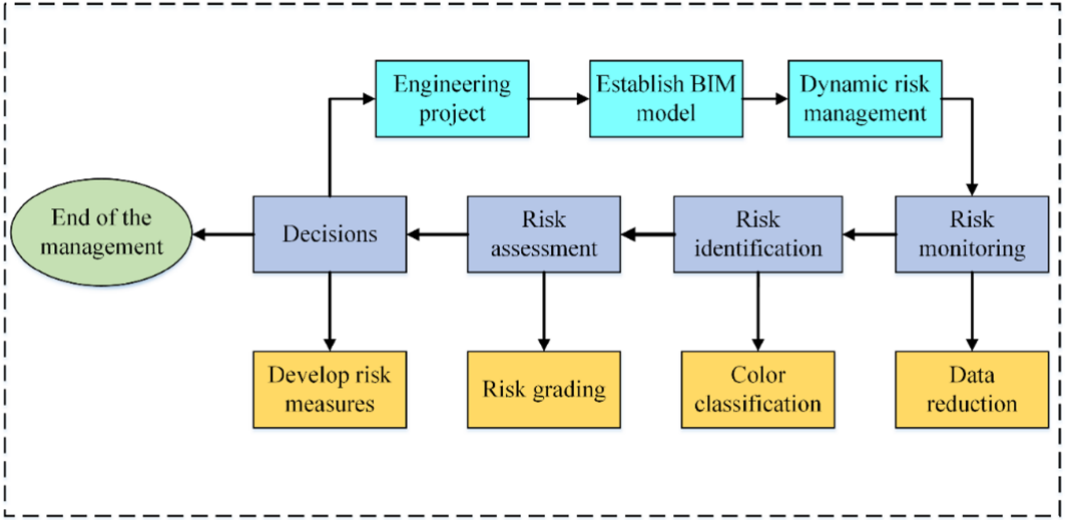
Dynamic risk management flowchart.

Tunnel engineering is strip engineering, along which the geological conditions are complicated and variable. Traditional risk management is simply based on the concept of “static risk” (assuming that the level of surrounding rocks in the tunnel and the stability of the tunnel face remain unchanged), and the corresponding risk plan is developed to ensure the safety of the tunnel construction. However, in the event of construction practices, many risk factors such as the level of surrounding rocks and the stability of the tunnel face may change with the construction progress, causing the actual situation, not in conformity with the original hypothesis. Therefore, it is indispensable to make reasonable risk plans based on the concept of “dynamic risk” management to reduce construction risks and ensure construction safety. Based on the above-mentioned digital management platform, combined with monitoring measurement data and advanced geological prediction data in the digital management platform, this paper proposes a construction risk management system based on BIM technology, which includes risk monitoring, risk identification and risk assessment and feedback. "Dynamic risk" management is the process of risk management as shown in Fig. 5. The risk management system can relieve the pressure for the analysis of supervision data. Technicians monitor tunnels by radar and total station, etc., and record information from the monitor. Then they can transfer the data to the database in time and quickly determine the health of the tunnel in the system. Finally, the system regularly sends the status of the tunnel to the relevant person in charge of email or Short Messaging Service.

### Risk monitoring

In terms of monitoring measurement, the first task is to classify the stability of the tunnel surrounding rocks. According to the existing classification criteria on the stability of surrounding rocks by evaluation indicators^53^, the stability of surrounding rocks can be divided into the following categories: Level I, which means steady, with the settlement deformation of 0–1 mm; Level II, which means relative steady, with the settlement deformation of 1–2 mm; Level III, which means almost steady, with the settlement deformation of 2–5 mm; Level IV, which means unsteady, with the settlement deformation of 5–10 mm; Level V, which means fairly unsteady, with the settlement deformation of 10–15 mm. Table 3 shows the classification criteria for the stability of surrounding rocks by evaluation indicators.

**Table 3:** Classification criteria on the stability of surrounding rocks by evaluation indicators.

Category	Steady I	Relative steady II	Almost steady III	Unsteady IV	Fairly unsteady V
Surrounding ground deformation (mm)	0–1	1–2	2–5	5–10	10–15

In the aspect of advanced geological prediction, this paper mainly studies the development degree of karsts, faults, fracture zones, and seepage of surrounding rock. According to the accuracy and reliability of the integrated advanced geological prediction method, this article will categorize the measured three kinds of the probability of the existence of bad geological conditions based on a comprehensive analysis of each advanced geological prediction result: “does not exist” is Level I, “small possibility to exist” is Level II, “large possibility to exist” is Level III, and “exist” is Level IV, among which Level I indicate best geological conditions and Level IV indicates worst geological conditions.

Based on the above classification of surrounding rock stability and geological development, this risk management system takes BIM technology as the link and adopts 3D visualization technology to describe the surrounding rock with different stability and poor geological conditions to different extents in the process of tunnel construction. According to the stability of the surrounding rock and the development degree of bad geological conditions, the construction managers can more accurately grasp the internal situation of the tunnel, which can lay a good foundation for the real-time adjustment and optimization of the construction plan.

The visualization scheme of BIM technology is as follows:

1. Visualization scheme for monitoring measurement
  It is required to establish the yellow model for the surrounding rocks with Level I (steady), the blue model for the surrounding rocks with Level II (relatively steady), the green model for the surrounding rocks with Level III (almost steady), orange model for the surrounding rocks with the Level IV (unsteady), and red model for the surrounding rocks with the Level V (fairly unsteady). Figure 6 shows the visualization scheme for the stability of surrounding rocks during tunnel monitoring measurement. When the level of the surrounding rock changes, the system will send out information to inform the management, so the condition of the surrounding rock should be taken seriously shortly. When the surrounding rock is in Level IV (unsteady) or Level V (fairly unsteady), the system will issue a warning to stop the construction.

**Figure 6:**
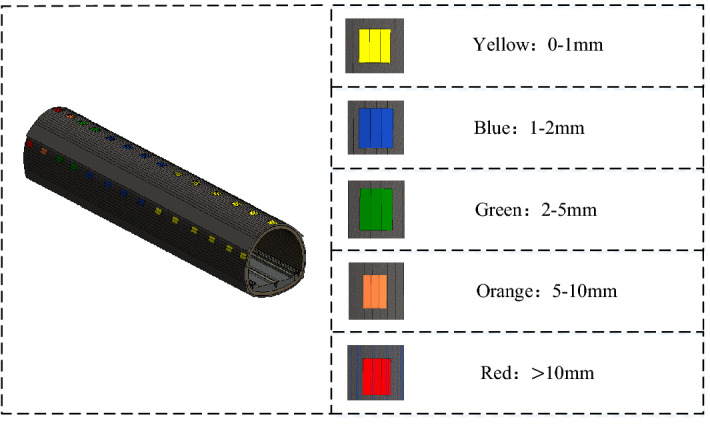
Visualization scheme diagram for the stability of surrounding rocks during tunnel monitoring measurement.
2. Visualization scheme for advanced geological prediction
  In terms of results gained through advanced geological prediction, “does not exist” is Level I for the karsts, faults, and fracture zones, and seepage of surrounding rock, shown in yellow, “small possibility to exist” is Level II, shown in orange, “large possibility to exist” is Level III, shown in blue, and “exist” is Level IV, shown in red.

### Risk identification

Risk identification is defined as the identification of security risks that may lead to casualties, environmental damage, economic loss, or project schedule delay. The key to the process of risk identification is to identify risk events, risk factors, the importance of risk factors, and their mutual relation. The stability of surrounding rocks in monitoring measurement and the development degree of the unfavorable geological body measured by tunnel geological prediction are directly related to the level of risk in tunnel construction. To facilitate risk management during construction, the risk in the tunnel construction is divided into eight levels: Level I, Level II, Level III, Level IV, Level V, Level VI, Level VII, and Level VIII. Among them, the risk degree of Level I risk is the lowest, and the risk degree of Level VIII risk is the highest.

1. Level I risk: The corresponding stability levels of surrounding rocks are "Level I-stable", "Level II-relatively stable", and "Level III-basically stable", and the result of advanced geological prediction is Level I;
2. Level II risk: The corresponding stability levels of surrounding rocks are "Level I-stable", "Level II-relatively stable", and "Level III-basically stable", and the result of advanced geological prediction is Level II;
3. Level III risk: The corresponding stability levels of surrounding rocks are "Level I-stable", "Level II-relatively stable", and "Level III-basically stable", and the result of advanced geological prediction is Level III;
4. Level IV risk: The corresponding stability levels of surrounding rocks are "Level I-stable", "Level II-relatively stable", and "Level III-basically stable", and the result of advanced geological prediction is Level IV;
5. Level V risk: The corresponding stability levels of surrounding rocks are "Level IV-unstable" and "Level V-extremely unstable", and the result of advanced geological prediction is Level I;
6. Level VI risk: The corresponding stability levels of surrounding rocks are "Level IV-unstable" and "Level V-extremely unstable", and the result of advanced geological prediction is Level II.
7. Level VII risk: The corresponding stability levels of surrounding rocks are "Level IV-unstable" and "Level V-extremely unstable", and the result of advanced geological prediction is Level III.
8. Level VIII risk: The corresponding stability levels of surrounding rocks are "Level IV-unstable" and "Level V-extremely unstable", and the result of advanced geological prediction is Level IV.

### Risk assessment

Based on a digital management platform, further risk assessment is necessary for the construction of the mountain tunnel, according to the results of risk monitoring and risk identification. The main purpose of the risk assessment is to determine the likelihood and impact of construction risks in the mountain tunnel and to formulate corresponding response measures so that effective action can be taken promptly when risks occur. The specific steps for implementing risk assessment are as follows:

1. Determine the likelihood of risks. By analyzing monitoring data, historical data, and advanced forecast data, and summarizing the experience of similar engineering projects and conditions, the likelihood of tunnel construction risks is analyzed.
2. Determine the impact level of risks. The impact level of risks is related to the nature of the risk event, the time and location of its occurrence, and so on. By observing the actual site conditions and analyzing monitoring data, the impact level of risks is assessed.
3. Determine the risk level: Combining the likelihood and impact level of risks, the risk level is determined. The eight levels of risks mentioned above are classified into four categories, namely low risk, medium risk, high risk, and very high risk. Specifically, level one and level two risks are low risk, level three and level four risks are medium risk, level five and level six risks are high risk, and level seven and level eight risks are very high risk.
4. Develop response strategies: Based on the determined risk level for the tunnel construction, corresponding response strategies are formulated. For high-level risks, more proactive and effective measures are needed to mitigate or eliminate the impact of risks, such as increasing monitoring frequency, formulating emergency plans, and taking remedial measures.
5. Establish a risk feedback mechanism: For the implementation and supervision of improvement measures proposed by the evaluation results, the process and measures of construction risk management are continuously optimized. Feedback from all parties is collected and responded to on time, to improve the management effectiveness and level.

The risk assessment results are stored in the management platform and fed back to all participating parties in the project, laying a foundation for the formulation of risk prevention plans. In addition, the risk levels will appear as the final result of the tunnel construction risk assessment in the project risk assessment report.

## Discussion

### The advantages of digital platform and dynamic risk management system

To observe the changes in tunnel monitoring measurement and advance forecast data more clearly, this paper puts forward the digital management platform architecture of tunnel engineering based on BIM technology from a new perspective. The digital platform and dynamic risk management system proposed in this paper have the following advantages:

1. The relevant tunnel engineering model established based on BIM technology contains all the component information of the tunnel, topographic and geological information, from which the relevant managers can quickly grasp various information of the tunnel, to arrange further work.
2. Automation of security risk identification process based on BIM, monitoring measurement, and advance forecast data are uploaded to the platform immediately after measurement, which saves a lot of time and human resources.
3. This study proposed the dynamic risk management system can replace traditional methods of security risks in the process of risk identification to reduce excessive dependence on experienced experts and engineers, and by using BIM visualization technology risk level can be reflected in the model, realize the field staff using the digital platform in mountain tunnel of risk management in the field of information sharing and integration.

### Feasibility analysis

Feasibility analysis levels are as follows:

1. Technical level: the proposed technology for the digital management platform architecture of tunnel engineering is monitoring measurement, advanced geological prediction, and BIM technology. Most of these technologies are very mature and have solid tech.
2. Functional level: the functional design of the platform is based on the unique characteristics of tunnel engineering and meets the actual needs of the tunnel engineering field. The proposed dynamic risk management system can replace traditional methods of security risks in the process of risk identification and realize the field staff using the digital platform in the mountain tunnel of risk management in the field of information sharing and integration.
3. Application level: tunnel engineering is strip engineering; the space span is large, and the geological conditions along the line are complex and changeable. The development of the platform is in line with the development trend of efficient construction and collaborative management of tunnel engineering and conforms to the development policy of the new era of the country.

## Conclusions

This paper introduces the combination of BIM technology and risk management in the field of mountain tunnel engineering for the first time. Based on this digital management platform, a set of construction risk management systems is proposed. Managers can effectively query the rock data of mountain tunnels in each construction stage and can avoid or resolve the risks arising from the construction, with the management system. This risk identification method can avoid excessive reliance on experts in this field. This digital platform can share and integrate risk management information. The research conclusions are as follows:

1. Based on BIM technology and combined with monitoring measurement and advanced geological prediction, which are two key parts in mountain tunnel construction, this paper preliminarily proposes the digital platform architecture for mountain tunnel construction. The platform is divided into the following three parts: basic layer, model layer, data layer, application layer, and user layer. The five layers are interrelated and correspond to each other to form a complete digital platform for mountain tunnel construction based on BIM technology, which is used for the collaborative management of all participants in mountain tunnel construction.
2. Based on the digital platform for mountain tunnel construction, this paper proposes a new set of relatively complete dynamic risk management systems for mountain tunnel construction based on BIM technology. This system is mainly divided into three key parts: risk monitoring, risk identification, and risk assessment. All parts correspond with each other to achieve efficient and dynamic risk management in mountain tunnel construction, reduce relative risk, increase construction safety, and ensure the construction safety of the whole construction process.
3. The digital platform and dynamic risk management system proposed in this paper have certain advantages in the construction of mountain tunnels and provide a new way for the management of safety risk in the construction of mountain tunnels, which has very important significance in the construction safety and risk control of mountain tunnel. In addition, the development of a scientific and reasonable risk warning theory and method is of great significance to the research of the entire digital risk platform construction process. Therefore, further research in this area is necessary for the continued improvement and advancement of the platform.

## Data Availability

All data generated or analyzed during this study are included in this published article.
